# Purchases of toddler formula in the USA: 17 years of household demographics and spending

**DOI:** 10.1017/S1368980026102055

**Published:** 2026-02-11

**Authors:** Jasmine H. Kaidbey, Janae T. Kuttamperoor, Andrea L. Sharkey, Erin A. Dowling, Teresa Conigliaro, Allison C. Sylvetsky

**Affiliations:** 1 Exercise and Nutrition Sciences, The George Washington University Milken Institute School of Public Health, USA; 2 New York City Department of Health and Mental Hygiene, USA

**Keywords:** Toddler milk, Toddler formula, Toddler drink, Sugary beverages, Child nutrition

## Abstract

**Objective:**

Toddler drinks, sometimes referred to as toddler milks or formulas, are not recommended by health authorities because they have a higher sugar and lower protein content than cows’ milk. However, advertisement spending and retail sales of these products have grown in the USA, and there is a nutrition surveillance gap in purchaser characteristics.

**Design:**

Household purchases of toddler drinks between 2004 and 2020.

**Setting:**

NielsenIQ consumer panel.

**Participants:**

Panellists across the USA.

**Results:**

Panellists purchased sixty-six unique toddler drinks between 2004 and 2020. Out of 202 207 households in the panel, 2644 panellists purchased toddler drinks at least once during the study period. Most panellists who purchased toddler drinks had a household income above $60 000 and had graduated from college. Households purchasing toddler drinks spent an average of $102 dollars, 1·5 % of their total food spending, on toddler drinks annually. The share of spending on toddler drinks increased by approximately 0·02 percentage points each year during the study period, which was equivalent to a 54 % increase between 2004 and 2020 (95 % CI: 0·04, 4 × 10^–3^). The highest average household spending on toddler drinks was among Asian households, households with a single male head of household and households with children 2–6 years old.

**Conclusions:**

Findings indicate that toddler drink purchasing patterns vary by household demographics and purchases have increased over time. Proactive efforts, including continued surveillance of toddler drink purchases and regulation of toddler drink marketing, are critical to promote consumption of age-appropriate beverages for young children.

The WHO recommends that mothers breastfeed their children until the children reach at least 2 years of age^(1)^ and introduce cows’ milk, or fortified soya beverages as a non-dairy alternative, when the children reach 6–36 months of age^(2)^. For women who cannot or choose not to breastfeed, the WHO and other health experts recommend Fe-fortified infant formula until the end of the first year after which cows’ milk is the recommended next beverage^(1–3)^. However, toddler drinks (also known as toddler milks, toddler formulas, growing-up milks and older infant-young child formulas)^(3)^ are widely advertised by manufacturers for consumption among 1- to 3-year-old children^(4)^. This advertising is concerning because toddler drinks generally have higher sugar and Na content as well as lower protein content compared to cows’ milk^(3,5–7)^, and the consumption of toddler drinks may contribute to excess caloric intake, which has been positively associated with later development of childhood obesity^(8)^. In addition, although toddler drinks do not offer nutritional benefits compared to cows’ milk, they are typically more expensive^(3,9,10)^.

While advertisements in the USA for infant formula have declined, those for toddler drinks have increased fourfold in recent years^(11)^, which raises the concern that manufacturers are increasingly focused on promoting toddler drinks^(7)^. From 2006 to 2015, total advertising on toddler drinks increased from less than $5 million annually to more than $20 million annually, while advertising on infant formulas peaked in 2010 and then declined^(11)^. From 2013 to 2015, toddler drink TV advertisements outpaced those for infant formula for the first time^(11)^. Television is the most common medium for advertising toddler drinks, but caregivers encounter additional exposure to advertising in print (e.g. magazines)^(5)^, social media, and at supermarkets and online retailers^(12)^.

Marketing practices that convey nutritional benefits may mislead parents into thinking that these products contain nutritional benefits not provided by food, or that they are healthier than cows’ milk, and may encourage them to purchase these products for their young children^(5,13)^. Toddler drink branding often resembles that of infant formula, which may make it difficult for parents to differentiate between infant and toddler products^(3,14)^ and promote the purchase of the ‘next step’ in the product line (i.e. toddler drinks after infant formula)^(3)^. Consistent with these concerns, dietary surveillance data in the USA indicate that some parents of infants younger than 6 months report providing ‘transition’ formulas intended for older infants, reflecting potential confusion of caregivers about age-appropriate beverages^(7,14)^. Furthermore, whereas infant formula is regulated in its composition^(3)^ and labelling^(15)^, there are no equivalent regulations for toddler drinks, leaving consumers at risk of being misled about the usefulness of these products^(15)^.

Early in the 1980s, in response to a decline in breast-feeding in many parts of the world and concerns that infant formula marketing was interfering with breast-feeding, the WHO published an ‘International Code of Marketing of Breastmilk Substitutes’^(16)^. The code aims to safeguard adequate nutrition for infants by protecting and promoting breast-feeding, as well as ensuring that breast milk alternatives are available for infants that need them^(16)^. In particular, the code’s provisions restrict the promotion of breast milk alternatives, including, for example, by recommending that manufacturers do not distribute breast milk alternatives as gifts or free samples, and similarly recommending that healthcare systems do not display breast milk alternatives. Although the USA did not adopt the WHO code, the Surgeon General supported its recommendations to hold marketers accountable for complying with the code in a 2011 call to action to support breast-feeding^(17)^. The American Academy of Pediatrics took a similar stance on toddler drinks in a 2023 report that stated that it does not recommend the use of toddler drinks over a diet with human or cows’ milk and solid foods rich in minerals and Fe, and that any marketing should make toddler drinks unambiguously distinct from infant formula, and retailers should not place these products on the same shelf^(3)^.

Along with the growing marketing of toddler drinks, increases in toddler drink sales have been reported worldwide^(5)^. However, prior analyses of toddler drink sales have mostly relied on retail data that lack demographic information about the purchasers^(11)^ or are based on surveys that asked caregivers to retrospectively report purchases of toddler drinks^(12,13,18)^. Prior research findings from small panel surveys suggest that Asian, Black and Hispanic/Latino consumers may be more likely to purchase and serve toddler drinks compared to non-Hispanic/Latino White individuals^(5)^, raising concerns that marketing practices could have a disproportionate impact on children from these backgrounds.

The objective of this study is to examine trends in purchases of toddler drinks over nearly two decades (2004–2020) and describe the sociodemographic characteristics of households that purchase toddler drinks in a large consumer panel from across the USA.

## Methods

### Data source

NielsenIQ is a global measurement and analytics company that collects consumer data, including retail spending^(19)^. The NielsenIQ consumer panel data, distributed by the Kilts Center for Marketing Data Center at The University of Chicago Booth School of Business, provides data from a consumer panel (recruited online via email and random site invitation) with 40 000–60 000 participants annually from around the USA. Panelists are required to be 18 years of age or older and consent to providing demographic information and reporting all their household purchases intended for personal, in-home use using a scanner or mobile app. Panellists are required to complete the survey of household demographic information annually, to reflect updates in dynamic household characteristics such as size, education, income, residence, age and marital status. For continued inclusion in the panel, NielsenIQ evaluates whether the household is meeting minimum reporting standards, which are based on household size; currently, 80 % of panellists are retained each year. Data from all NielsenIQ panellists and all panel years available at the time of this investigation (July 2023) were used.

### Identifying unique universal product codes to include in analytic dataset

Between 2004 and 2020, there were 2742 universal product codes (UPC) for ‘baby milk and milk flavoring’ within the NielsenIQ products master file. Of these, eighty-eight UPC were classified within NielsenIQ as ‘toddler formula’ or ‘infant & toddler formula’ and identified as potentially eligible for our analysis. The distinction between these two categories is that ‘infant and toddler formulas’ are labelled for use by both infants (< 12 months) and toddlers (12 months and older), whereas toddler drinks are specifically for toddlers 12 months or older. An example of an ‘infant and toddler formula’ is Gerber Good Start 2, which is labelled for use by children aged 9–24 months old. Of the eighty-eight UPC, eleven were excluded because their category could not be determined (possibly due to private-label store brands)^(11)^, and eleven were excluded because they were infant formulas that were not also for toddlers. Thus, a total of sixty-six UPC were identified as ‘toddler formula’ or ‘infant and toddler formula’ and included in the analysis. Hereafter, both ‘infant and toddler formula’ and ‘toddler formula’ will be collectively referred to as toddler drinks. To calculate total food and beverage spending, UPC of any food products were identified by including all food and beverage products in the NielsenIQ food categories (dairy products, deli, dry grocery, fresh produce, frozen foods or packaged meat), except alcoholic beverages.

### Statistical analysis

Panellists that purchased a toddler drink were identified based on having at least one UPC for toddler drinks in their purchase history over the study period, and their demographic characteristics were examined using frequencies and summarised as percentages. Toddler drink purchases were examined both in terms of the number of products purchased and household spending in USA Dollars (USD). To control for inflation, amounts spent were converted into 2020 USD by multiplying the dollar amount spent in any given year by the consumer price index for 2020 and then dividing by the consumer price index for the given year^(20)^.

### Trends in toddler drink purchases

Trends in the number of unique toddler drink products purchased were examined by year. To test this trend, the number of UPC purchased were summed for each year, and linear regression was used to test if there was a linear trend in product number by year. Linear regression assumptions were verified: residuals of the model followed a normal distribution based on the Anderson–Darling test (A = 0·40, *P* = 0·3), and there was no evidence of heteroskedasticity based on the Breusch–Pagan test (BP = 0·12, *P* = 0·72).

Next, trends in toddler drink spending were analysed using linear mixed models – an analytic approach that could account for the repeated measurement of household-level purchases. To quantify spending, the amount spent by each household on toddler drinks per year was summed. Toddler drink spending was also expressed as a percentage of all food and beverage purchases. Spending on toddler drinks divided by total food and beverage spending is hereafter referred to as the share of food spending on toddler drinks and was expressed as a percent.

### Characteristics of toddler drink purchasers

To examine associations between participant demographics and spending, total household spending on toddler drinks per year and share of total food spending on toddler drinks per year were calculated and analysed as separate linear mixed models for each subgroup of interest: age, race, Hispanic origin (ethnicity), income, educational attainment, head of household structure (male, female or dual), and age and number of children in the household. For panellists who participated in the panel over multiple years, demographic characteristics reported in the first year they purchased toddler drinks were used in the analysis. Sensitivity analyses were conducted for characteristics that were significantly associated with spending, to address whether there may have been confounding by income, age and head of household structure. Adjusted R^2^ were also presented to assess the explained variance by the confounder-adjusted models.

All analyses used sample weights derived by NielsenIQ that are used to balance the unweighted panel according to the following demographics of USA households: size, income, head of household age, education, occupation, race, ethnicity, presence of children and county size. One adult per household is designated as the NielsenIQ panellist and reports their demographic characteristics (e.g. race) and the household income. Age, sex and education are specified for each adult head of household. All analyses were conducted using RStudio, and a two-sided *P*-value of < 0·05 was used for statistical significance^(21)^.

## Results

Between 2004 and 2020, the NielsenIQ consumer panel included 202 207 participating households, of which 2644 purchased toddler drinks at least once over the 17-year period (see online supplementary material, Supplemental Table 1). Using sample weights to reflect characteristics of USA households, the panellists were projected to reflect about 8·5 million households that purchased toddler drinks over the study period (Table 1). The number of households that purchased toddler drinks varied by year, with the total number across the USA estimated to range from 110 441 to 1 255 256. Whereas only 20 % of all panellists had a child under 6 years old, most panellists who purchased toddler drinks had a child under 6 years of age in the household (67 %). Similarly, the prevalence of households without children was 55 % in the full panel, compared with 24 % in the subsample of households that purchased toddler drinks. Most panellists that purchased toddler drinks were between the ages of 25 and 44 years, had a household income above $60 000, lived in two heads of household and had graduated from college.

**Table 1.** Demographic characteristics of toddler drink purchasers and all panellists between 2004 and 2020 in the NielsenIQ consumer panel

	Toddler drink purchasers (in thousands)		All panellists (in thousands)	
	8559		529 851	
	*n*	%	*n*	%
Age of female head of household^*^				
< 25	223	2·7 %	10 264	2·3 %
25–34	5179	62·5 %	149 546	33·6 %
35–44	1595	19·3 %	99 338	22·3 %
45–54	705	8·5 %	98 937	22·2 %
55+	580	7 %	86 790	19·5 %
Age of male head of household^*^				
< 25	59	0·8 %	5659	1·5 %
25–34	3632	48·7 %	98 231	26 %
35–44	2516	33·8 %	103 097	27·3 %
45–54	701	9·4 %	87 461	23·1 %
55+	545	7·3 %	83 864	22·2 %
Head of household				
Female and male	7175	83·8 %	293 337	55·4 %
Female only	1106	12·9 %	151 538	28·6 %
Male only	277	3·2 %	84 976	16 %
Ethnicity				
Non-Hispanic	1558	18·2 %	72 157	13·6 %
Hispanic	7001	81·8 %	457 694	86·4 %
Race				
White/Caucasian	6322	68 %	398 408	75·2 %
Black/African American	1125	12·1 %	64 104	12·1 %
Asian	769	8·3 %	18 955	3·6 %
Other	1084	11·7 %	48 384	9·1 %
Educational attainment of female head of household				
< High school	276	3·2 %	20 136	3·8 %
High school graduate	1471	17·2 %	146 824	27·7 %
Some college	2537	29·6 %	181 647	34·3 %
College graduate or more	4276	50 %	181 243	34·2 %
Educational attainment of male head of household				
< High school	373	4·4 %	33 152	6·3 %
High school graduate	1977	23·1 %	159 062	30 %
Some college	2766	32·3 %	170 600	32·2 %
College graduate or more	3444	40·2 %	167 037	31·5 %
Family income				
Under $19 999	513	6·0 %	79 278	15 %
$20 000–$39 999	1286	15·0 %	126 567	23·9 %
$40 000–$59 999	1866	21·8 %	102 496	19·3 %
$60 000–$69 999	617	7·2 %	37 317	7 %
$70 000–$99 999	2122	24·8 %	92 974	17·5 %
$100 000+	2154	25·2 %	91 219	17·2 %
Ages of children in the household				
Children under 6^*^	5697	66·6 %	107 237	20·2 %
Under 2	3449	40·3 %	15 124	2·9 %
Only children over 6	804	9·4 %	132 424	25 %
No children under 18	2057	24·0 %	290 190	54·8 %
Children in the household^†^				
1	2994	46 %	104 145	43·5 %
2	2041	31·4 %	85 285	35·6 %
3	1062	16·3 %	35 647	14·9 %
4 or more	405	6·2 %	14 593	6·1 %

All values are weighted using NielsenIQ sample weights based on Census data for demographic characteristics of households in the USA.

* Households with children under 6 years of age may also have older children.

† Among households with children under 18 years of age.

**Figure 1.**
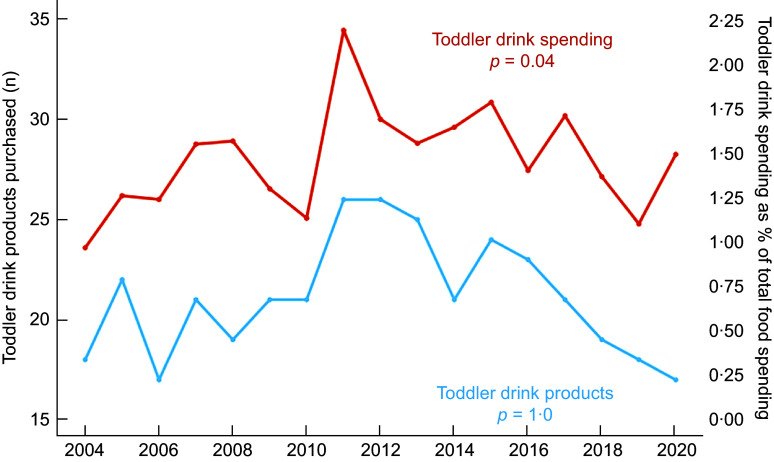
Number of toddler drink products purchased (blue) and toddler drinks spending (orange) as a percent of total food spending among NielsenIQ consumer panel participants from 2004 to 2020. Spending values were in 2020 USD and weighted to reflect nationally representative household characteristics. Toddler drink products (primary y-axis) reflect the number of distinct toddler products purchased by panellists, by year. Toddler drink spending (secondary y-axis) reflects the percent of a household’s total food spending devoted to toddler drink purchases, averaged across all households each year.

The number of unique toddler drink products purchased within a given panel year ranged from 17 to 26. No significant differences in the total number of toddler drink products purchased over time were observed (*β* < 0·001, se: 0·15, *P* = 1·0, Figure 1). However, there was an increase in spending on toddler drinks as a percent of total food spending (*β* = 0·02, 95 % CI: 0·04, 4 × 10^–3^%, *P* = 0·04, Figure 1). Compared to 2004, where spending on toddler drinks was 0·97 percent of total food spending, the share increased to 1·49 % in 2020 – indicating a 54·2 % increase. Toddler drink spending was, on average, 1·48 % of households’ total food spending over the course of the study period (95 % CI: 1·34 %, 1·61 %), or about $102·3 dollars per household (95 % CI: $89·19, $115·38).

As shown in Table 2, total household spending varied by several demographic characteristics. Although Asian households only accounted for 8·3 % of panellists who purchased toddler drinks, this subgroup accounted for 12·6 % of total spending on toddler drinks (Supplemental Table 2). Compared to White households, Asian households spent about $48·6 more per year on toddler drinks (95 % CI: $30·6, $66·6), and 1·03 percentage points more in the share of their total food spending on toddler drinks (95 % CI: 0·76 %, 1·3 %). Households with older children or without children spent significantly less per year on toddler drinks, compared to households with children aged 2 years or younger. The number of children in the household was not associated with total spending on toddler drinks; however, households with three or more children spent a smaller share of their food spending on toddler drinks than homes with one child (*β*: –0·23 %, 95 % CI: –0·42 %, –0·05 %). The share of household food spending among households headed by a single male was 0·75 percentage points greater than dual-headed households (95 % CI: 0·16 %, 1·35 %). Among male heads of household, age was not associated with total spending on toddler drinks or the share of their food spending on toddler drinks; however, for female heads of household, those 45–54 years old spent 0·71 percentage points less than females under 25 years of age. Income, education level, ethnicity and age of the male head of the household were not associated with total spending.

**Table 2.** Household spending on toddler drinks stratified by demographic characteristics of NielsenIQ consumer panel participants between 2004 and 2020, in 2020 inflation-adjusted US Dollars (USD)

	Average annual spending by household		Share of annual food spending	
	*β* coefficient	95 % CI	*β* coefficient	95 % CI
Age of female head of household				
< 25	*Reference*		*Reference*	
25–34	−28·0	–154·8, 98·7	0·12	–0·48, 0·71
35–44	13·0	–108·8, 134·8	0·05	–0·55, 0·65
45–54	−18·8	–140·8, 103·2	**−0·61**	**–1·04, –0·19**
55+	−19·7	–140·7, 101·2	−0·46	–1·07, 0·16
Age of male head of household				
< 25	*Reference*		*Reference*	
25–34	31	–35·2, 97·2	0·09	–0·91, 1·08
35–44	38·1	–28, 104·1	0·01	–0·99, 1·01
45–54	17	–50·1, 84·1	−0·45	–1·46, 0·56
55+	−4·7	–71·5, 62	−0·61	–1·62, 0·39
Head of household				
Female and male	*Reference*		*Reference*	
Female only	**−20**	**–36·5, –3·5**	−0·15	–0·4, 0·11
Male only	−8·8	–47·7, 30·1	**0·75**	**0·16, 1·35**
Ethnicity				
Non-Hispanic	*Reference*		*Reference*	
Hispanic	0·49	–15·4, 16·4	−0·01	–0·25, 0·23
Race				
White/Caucasian	*Reference*		*Reference*	
Black/African American	1·8	–14·9, 18·5	**0·3**	**0·04, 0·55**
Asian	**48·6**	**30·6, 66·6**	**1·03**	**0·76, 1·3**
Other	15·5	–2·7, 33·8	**0·35**	**0·07, 0·63**
Educational attainment^*^				
< High school	*Reference*		*Reference*	
High school graduate	−30·7	–70·2, 50·6	−0·31	–0·77, 0·07
Some college	−13·55	–52·0, 24·9	−0·13	–0·54, 0·27
College graduate or more	−14·88	–52·7, 22·9	−0·07	–0·47, 0·34
Family income				
Under $19 999	*Reference*		*Reference*	
$20 000–$39 999	1·7	–26·2, 29·6	−0·03	–0·46, 0·39
$40 000–$59 999	8·8	–17·9, 35·5	0·03	–0·38, 0·44
$60 000–$69 999	−2	–31·4, 27·4	0·01	–0·44, 0·46
$70 000–$99 999	−3·6	–30·1, 23	−0·23	–0·63, 0·18
$100 000+	13·6	–13·5, 40·8	0·05	–0·37, 0·46
Ages of children in the household				
Under 2	*Reference*		*Reference*	
> 2–under 6^†^	6·6	–7·3, 20·5	0·04	–0·18, 0·25
Only children over 6	**−29·4**	**–48, –10·8**	**−0·33**	**–0·61, –0·04**
No children under 18	**−27·3**	**–39·5, –15·2**	**−0·31**	**–0·5, –0·13**
Children in the household^‡^				
1	*Reference*		*Reference*	
2	−0·9	–15·6, 13·8	0·03	–0·13, 0·19
3 or more	−12·6	–29·7, 4·5	**−0·23**	**–0·42, –0·05**

Household spending was characterised as the annual average toddler drink purchases by households and as the annual share of total household food spending on toddler drinks. Values for the household spending are presented as *β* coefficients from linear mixed models, where each demographic characteristic was used as a predictor of spending. All values are weighted using NielsenIQ sample weights based on Census data for demographic characteristics of households in the USA. Bolded values indicate *P* < 0·05.

* Estimates were not significantly different by education of the male v. female head of household for households with dual heads. Results are presented with the female head of household’s education.

† Households with children under 6 years of age may also have older children.

‡ Among households with children under 18 years of age.

Sensitivity analyses reanalysed variables that were significantly associated with spending (sex of the head of household, age of the female head of household, race and the age and number of children) in a multivariable model that controlled for income (see online supplementary material, Supplemental Table 3). Although income itself was not significantly predictive of spending, it may still be associated with race and confound the relationship between race and spending. Adjusted analyses continued to find that Asian households were predictors of higher spending than White households ($46·48, 95 % CI: 28·47, 64·50 and 0·49 %, 95 % CI: 0·30, 0·68), as did having a single male head of household rather than a dual head of household (0·62 %, 95 % CI: 0·21, 1·02). After adjustment, total spending by female-headed households compared with dual-headed households was no longer significant ($-7·12, 95 % CI: –25·2, 11·0). Furthermore, spending in households without children, was no longer significantly less than in households with children under 2 years of age. The marginal R^2^ estimates (i.e. accounting only for the fixed effects of the demographics and not the random effects of households) explained 4 % of the variance in spending on toddler drinks. The variance explained by both fixed and random effects (i.e. conditional R^2^ estimates) was 28 % for total spending and 34 % for the share of spending on toddler drinks.

## Discussion

In a national consumer panel in the USA, the share of total food spending on toddler drinks increased by 54 % between 2004 and 2020 and was not explained by households purchasing a larger number of products, but rather, households spending more on a similar number of products. Previous research has shown an increase in advertising and sales of toddler drinks over a similar period (2006–2015)^(11)^, as well as an increase in total TV advertising of children’s drinks^(22)^. Although increased spending may be due to increased marketing of these products^(11)^, the present findings did not model advertising and spending simultaneously and cannot establish causality. Nevertheless, qualitative research has found that caregivers mistakenly believe that toddler drinks offer nutritional benefits beyond cows’ milk^(13)^ – which could be in part due to claims from toddler drink advertising. Experts from the Academy of Pediatrics explain that this mistaken belief of caregivers may be partly accounted for by a misinterpretation of structure-function claims – claims that product ingredients support body functions but do not need to be based on scientific evidence about the product^(3)^. Toddler drinks have been estimated to have an average of four nutrition-related claims per package, providing ample potential for caregivers to attribute unproven nutritional value to these products^(7)^. In addition to the misleading claims included on labelling and packaging, TV advertising for toddler drinks has included phrases like ‘since 85 % of brain growth is complete by age three… now is the time to nourish them’, which could persuade caregivers concerned about their children’s optimal growth to purchase products^(7)^. Other research on children’s beverage intake more broadly has shown misalignment in parents’ perceptions of beverages and public health guidance. For example, qualitative research on parents’ perceptions of healthy toddler drinks has found that parents may be surprised to learn that beverages marketed with added vitamins may still contain high amounts of sugar and are not recommended^(23)^. Likewise, parents of toddlers have been found to prioritise purchasing products that are marketed as ‘natural’ or containing nutrients such as *n*-3s^(24)^.

Findings of the present analysis are similar to some prior findings examining toddler drink purchases by race and ethnicity^(5)^. For example, previous studies have found that Asian and Black caregivers were more likely to buy toddler drinks compared to caregivers from other racial/ethnic backgrounds^(5)^. Similarly, households that identified as Asian, Black or a race other than those listed were overrepresented among households that purchased toddler drinks, and, also had a greater share of their food spending on toddler drink purchases relative to their White counterparts, as previously described in the literature^(13)^. Consistent with prior findings, the present analysis found households that purchased toddler drinks had higher income compared to the overall sample of panellists^(5)^, although differences in spending by household income were not statistically significant in the present investigation. This may reflect the limited variability in income among purchasers (half of the sample fell into the two highest income categories). Similarly, education was not significantly associated with spending, and about 80 % of the purchasers of toddler drinks had at least some college education. The absence of significant associations may also be a product of self-selection into the analytic sample by caregivers who perceive the products as beneficial and a broad reach of marketing that cuts across socio-economic groups.

The finding that male-headed households had higher spending on toddler drinks compared to dual-headed households is noteworthy and, to our knowledge, has not been reported in prior studies. Previous work has shown that higher earners tend to spend more on toddler drinks^(5)^; this could partially explain this finding since, on average, single fathers earn more than single mothers^(25)^. Another noteworthy finding was that throughout the 17-year study period, 24 % of all toddler beverage purchases were by households that did not have a child under 6 years of age in the home. One possible explanation is that these panellists are relatives of young children, such as grandparents, who purchase the drinks for children outside their household. Other possibilities are that the demographic survey was completed when a member of the household was pregnant and purchased toddler drinks before the infant was born, that they were purchased by parents not living in the same household as their toddlers, or that the beverages are being consumed by older children.

This study had several important strengths. A key strength of the NielsenIQ household consumer panel data are that they contain at least one year of purchasing data from each household, as compared to previous studies that asked survey participants about purchases in the last month^(13,18)^ or collected a retrospective estimate of how many times they ever purchased toddler drinks^(12)^. Furthermore, this analysis includes nationally representative survey weights that allow for the projection of spending estimates to all USA households; it also accounts for inflation over the 17-year period. Several limitations of this study also warrant consideration. First, estimates of spending on toddler drinks are subject to measurement error due to the method NielsenIQ uses to assign product prices. For retailers that are not included in the NielsenIQ retailer network (approximately 50 % of USA sales), panellists report the price paid, whereas NielsenIQ reduces the burden on its panellists by using the national average weekly price for corresponding UPC when products are purchased at large retailers^(26)^. Next, the dataset precludes the ascertainment of the exact ages of the children within the household or the determination of which members of the household consumed the toddler drinks. NielsenIQ provides the birth year of each member within the panellist household, but that only allows for rough estimation of the child’s age (by subtracting panel year minus birth year), and it is therefore not possible to distinguish between infants (0–1) and young toddlers (1–2) in the dataset.

Prior studies using other data sources (i.e. not NielsenIQ) have investigated purchases of toddler drinks by the ages of children consuming them, including one that determined caregivers are buying toddler drinks for infants younger than 12 months, for whom, toddler drinks are not a recommended, suitable alternative to breast-feeding or infant formula^(5)^. That study also found that Black caregivers were more likely to purchase toddler beverages for infants 6–11 months old compared to caregivers from other racial backgrounds, despite these beverages not being formulated to provide the key nutrients during this critical period of development^(18)^. While this could not be addressed in the present study due to the limited available information about the age of children in the household, the extent to which toddler drinks are inadvertently consumed by infants warrants further investigation.

Increased spending on toddler drinks, despite not being recommended by paediatric experts, raises important concerns. Toddlerhood is a critical period for physical growth and dietary expansion. Increased spending on toddler drinks points to the need for policy solutions to protect consumers who may be unaware that toddler drinks are not recommended^(3)^. Pediatric health authorities^(3)^ and legal analyses have called for ‘clear, transparent, and accurate’ labelling^(15)^ of toddler drinks and put forth several recommendations, including discontinuation of the often similar branding for infant formula and toddler drinks, and efforts to avoid linking infant formula to toddler drinks to prevent consumers from assuming that toddler drinks are needed following discontinuation of infant formula. The federal government in the USA has an opportunity to take action via the Federal Trade Commission to challenge misleading toddler drink marketing^(11,27)^ and to invest in an information campaign for parents and caregivers on why toddler drinks are not recommended, much like former promotional campaigns launched by the US Departments of Agriculture and Health and Human Services to support breast-feeding^(17,28)^. If the Food and Drug Administration (FDA) pursues added sugars targets in packaged food products and implements front-of-package warning labels for added sugars, toddler drinks should explicitly be included despite the proposed FDA exclusion of products marketed for infants and children aged 1–3 years^(29)^. Furthermore, toddler drinks could also be regulated to prevent them from being displayed alongside infant formulas or branded with the use of child development stages^(27,30)^ that imply that they are recommended after breast milk or medically necessary breast milk alternatives. Governments could also implement excise taxes on these products that could generate revenue for public health programmes^(30)^. Lastly, paediatricians should not implicitly endorse these products by displaying them in their clinics^(17)^, and they can educate patients on appropriate beverage choices during the transition from breast-feeding and formula use^(3)^.

### Conclusions

In this study of USA households, panellists spent about 100 dollars per year on toddler drinks, or about 1·5 % of their total food spending – a 54 % increase over the 17-year period studied. This quantity of spending on toddler drinks is not trivial, especially given that it may divert resources from more nutritionally dense foods and beverages and potentially contribute to excess sugar and Na intake. Continued surveillance of toddler drink purchases is critical to assess changes in the demand for and use of these products over time. Future research should also investigate additional key drivers of toddler drink purchases in tandem with the studied sociodemographic characteristics – such as marketing exposure and toddler feeding practices.

## Supporting information

Kaidbey et al. supplementary material Kaidbey et al. supplementary material

## Supplementary material

For supplementary material accompanying this paper visit https://doi.org/10.1017/S1368980026102055
