# Supplementary material for: Purchases of toddler formula in the USA: 17 years of household demographics and spending
Source: Public Health Nutr. 2026 Feb 11;29(1):e49. doi: 10.1017/S1368980026102055 (PMC12979013; doi:10.1017/S1368980026102055)
Supplement: Kaidbey et al. supplementary material [file S1368980026102055sup001.docx]

**Supplemental table 1.** Unweighted demographic characteristics of toddler drinks purchasers and all panelists between 2004-2020 in the NielsenIQ consumer panel.

|  | **Toddler Drink Purchasers, n (%)** | **All Panelists, n (%)** |
| --- | --- | --- |
| **n** | 2,644 (100%) | 202,207 (100%) |
| **Age of Female Head of Household**  <25  25- 34  35- 44  45- 54  55+ | 44 (1.7%)  1019 (39.2%)  768 (29.6%)  340 (13.1%)  426 (16.4%) | 2179 (1.2%)  30503 (16.4%)  46964 (25.3%)  49972 (26.9%)  55930 (30.1%) |
| **Age of Male Head of Household**  <25  25- 34  35- 44  45- 54  55+ | 15 (0.6%)  761 (32.2%)  849 (35.9%)  322 (13.6%)  416 (17.6%) | 1176 (0.8%)  21457 (14%)  39113 (25.5%)  40781 (26.5%)  51136 (33.3%) |
| **Head of Household**  Female only  Male only  Female and Male | 281 (10.6%)  47 (1.8%)  2316 (87.6%) | 48544 (24.0%)  16659 (8.2%)  137004 (67.8%) |
| **Ethnicity**  Hispanic  Non-Hispanic | 300 (11.3%)  2344 (88.7%) | 15206 (7.5%)  187001 (92.5%) |
| **Race**  White/Caucasian  Black/African American  Asian  Other | 1931 (73%)  275 (10.4%)  218 (8.2%)  220 (8.3%) | 163864 (81%)  20477 (10.1%)  6321 (3.1%)  11545 (5.7%) |
| **Educational attainment**  < High School  High School Grad  Some College  Graduated College  Post College Grad | 67 (2.5%)  427 (16.1%)  772 (29.2%)  986 (37.3%)  392 (14.8%) | 7156 (3.5%)  45495 (22.5%)  63876 (31.6%)  61896 (30.6%)  23784 (11.8%) |
| **Family income**  Under $19,999  $20,000-$39,999  $40,000-$59,999  $60,000-$69,999  $70,000-$99,999  $100,000+ | 113 (4.3%)  406 (15.4%)  641 (24.2%)  256 (9.7%)  704 (26.6%)  524 (19.8%) | 21007 (10.4%)  49364 (24.4%)  46389 (22.9%)  18072 (8.9%)  38951 (19.3%)  28424 (14.1%) |
| **Ages of children in the household**  Children under 6^*^  *Under 2*  Only children over 6  No Children <18 | 1440 (54.5%)  *851 (32.2%)*  244 (9.2%)  960 (36.3%) | 28762 (14.2%)  *3801 (5.3%)*  43669 (21.6%)  129776 (64.2%) |
| **Children** in the household ^†^  1  2  3  4 or more | 779 (46.3%)  543 (32.2%)  250 (14.8%)  112 (6.6%) | 31760 (43.8%)  26562 (36.7%)  9976 (13.8%)  4134 (5.7%) |

^*^ Households with children under 6 may also have older children

^†^ Among households with children under 18

**Supplemental table 2.**  Total toddler drink spending by household characteristics in the NielsenIQ consumer panel, in 2020 Inflation-Adjusted United States Dollars (USD). All values are weighted using NielsenIQ sample weights based on Census data for demographic characteristics of households in the United States.

| **Characteristic** | Total spending (95% Confidence Interval) by Demographic Characteristic, 2020 USD in Thousands. |
| --- | --- |
| **Total sample** | 1,024,250 (873,980 – 1,174,521) |
| **Age of Female Head of Household** |  |
| <25 | 2,551.0 (34.0 – 5,068.0) |
| 25- 34 | 13,166.9 (6,732.7 – 19,601.1) |
| 35- 44 | 45,146.3 (18,683.1 – 71,609.5) |
| 45- 54 | 22,126.6 (9,922.1 – 34,331.1) |
| 55+ | 15,545.2 (3,914.0 – 27,176.4) |
| **Age of Male Head of Household** |  |
| <25 | 5,225.2 (1,081.6 – 9,368.8) |
| 25- 34 | 417,452.9 (361,786.4 – 473,119.5) |
| 35- 44 | 324,144.2 (250,868.9 – 397,419.5) |
| 45- 54 | 127,048.5 (9,859.4 – 244,237.6) |
| 55+ | 51,843.5 (32,688.1 – 70,998.9) |
| **Head of Household** |  |
| Female and Male | 922,075.98 (770,386.04, 1,073,765.92) |
| Female only | 98,958.01 (66,615.53, 131,300.49) |
| Male only | 27,577.11 (13,272.47, 41,881.74) |
| **Ethnicity** |  |
| Non-Hispanic | 841,887.5 (704,844.1 – 978930.9) |
| Hispanic | 182,362.8 (117,197.1 – 247,528.5) |
| **Race** |  |
| White/Caucasian | 661,202.4 (530,854.5 – 791,550.4) |
| Black/African American | 87,346.8 (67,735.7 – 107,721.4) |
| Asian | 131,878.7 (83,540.9 – 171,314.3) |
| Other | 150,943.3 (83,957.3 – 211,826.2) |
| **Educational attainment of Female Head of Household** |  |
| < High School | 27,411.5 (11,242.3 – 43,580.6) |
| High School Graduate | 195,322.1 (74,506.5 – 316,137.6) |
| Some College | 322,171.7 (247,136.2 – 397,207.2) |
| College Graduate or more | 479,345.1 (423,879.4 – 534,810.8) |
| **Educational attainment of Male Head of Household** |  |
| < High School | 29,710.0 (13,762.9 – 45,657.2) |
| High School Graduate | 289,862.4 (163,827.0 – 415,897.9) |
| Some College | 326,825.7 (256,819.8 – 396,831.6) |
| College Graduate or more | 326,825.7 (256,819.8 – 396,831.6) |
| **Family income** |  |
| Under $19,999 | 57,530.4 (29,219.7– 85,841.0) |
| $20,000-$39,999 | 145,322.3 (83,956.6 – 206,688.0) |
| $40,000-$59,999 | 264,998.0 (142,524.0 – 387,471.9) |
| $60,000-$69,999 | 79,765.3 (55,764.5 – 103,766.1) |
| $70,000-$99,999 | 233,968.6 (193,723.6 – 274,213.7) |
| $100,000+ | 242,665.8 (197,597.2 – 287,734.4) |
| **Ages of children in the household** |  |
| Under 2 | 402,393.4 (329,557.0 – 475,229.9) |
| > 2 - Under 6^†^ | 282,461.7 (229,859.0 – 335,064.5) |
| Only children over 6 | 75,676.1 (45,783.8 –105,568.4) |
| No Children under 18 | 263,719.0 (142,560.0 – 384,878.1) |
| **Children in the household**^†^ |  |
| 1 | 419,748.0 (320,301.9 – 519,194.1) |
| 2 | 276,841.7 (230,489.5 – 323,193.9) |
| 3 or more | 143,827.2 (115,736.4 – 171,918.0) |

^† †^Households with children under 6 may also have older children

^†^ Among households with children under 18

**Supplemental table 3.**  Multivariable models of the associations between demographic characteristics and household spending (annual average toddler drink purchases by household, lefthand side, and annual share of total household food spending, righthand side). Data are from NielsenIQ consumer panel participants between 2004-2020, in 2020 Inflation-Adjusted United States Dollars (USD). Beta coefficients (95% Confidence Interval) from linear mixed models are presented. Bolded values indicate p<0.05.

| Characteristic | Average annual spending by household | Share of annual food spending on toddler drinks |
| --- | --- | --- |
| Head of Household |  |  |
| Female and Male | Reference | Reference |
| Female only | -7.12 (-25.2- 11.0) | -0.01 (-0.201, 0.182) |
| Male only | -5.46 (-44.09, 33.17) | **0.616 (0.208, 1.024)** |
| Age of Female Head of Household* |  |  |
| <25 | Reference | Reference |
| 25- 34 | 26.46 (-12.89, 65.81) | 0.001 (-0.416, 0.417) |
| 35- 44 | 36.57 (-3.25, 76.39) | -0.058 (-0.48, 0.363) |
| 45- 54 | 36.57 (-3.25, 76.39) | **-0.453 (-0.891, -0.014)** |
| 55+ | 9.95 (-31.39, 51.30) | -0.273 (-0.71, 0.164) |
| Race |  |  |
| White/Caucasian | Reference | Reference |
| Black/African American | 4.94 (-11.94, 21.82) | 0.174 (-0.004, 0.353) |
| Asian | **41.09 (23.02, 59.16)** | **0.491 (0.299, 0.683)** |
| Other | 17.95 (-2.62, 38.53) | 0.15 (-0.042, 0.342) |
| Family income |  |  |
| Under $19,999 | Reference | Reference |
| $20,000-$39,999 | -4.79 (-32.64, 23.06) | 0.012 (-0.282, 0.306) |
| $40,000-$59,999 | -2.86 (-30.01, 24.28) | 0.013 (-0.274, 0.3) |
| $60,000-$69,999 | -14.14 (-44.11, 15.83) | -0.015 (-0.332, 0.302) |
| $70,000-$99,999 | -16.79 (-44.12, 10.53) | -0.162 (-0.451, 0.126) |
| $100,000+ | -4.36 (-32.42, 23.69) | -0.092 (-0.389, 0.204) |
| Ages of children in the household |  |  |
| Under 2 | Reference | Reference |
| > 2 - Under 6* | 3.64 (-10.16, 17.45) | 0.002 (-0.145, 0.148) |
| Only children over 6 | **-27.85 (-47.01, -8.69)** | **-0.231 (-0.434, -0.028)** |
| No Children under 18 | 22.43 (-3.67, 48.53) | 0.218 (-0.055, 0.491) |
| Children in the household ^†^ |  |  |
| 1 | Reference | Reference |
| 2 | -3.78 (-17.30, 9.75) | 0.003 (-0.138, 0.145) |
| 3 or more | **-16.38 (-32.32, -0.43)** | **-0.193 (-0.36, -0.025)** |
| Marginal Adjusted R^2^ | 0.04 | 0.04 |
| Conditional Adjusted R^2^ | 0.28 | 0.34 |

^*^Households with children under 6 may also have older children.

^†^ Among households with children under 18
